# Study protocol of the LARK (TROG 17.03) clinical trial: a phase II trial investigating the dosimetric impact of Liver Ablative Radiotherapy using Kilovoltage intrafraction monitoring

**DOI:** 10.1186/s12885-021-08184-x

**Published:** 2021-05-03

**Authors:** Yoo Young Dominique Lee, Doan Trang Nguyen, Trevor Moodie, Ricky O’Brien, Anne McMaster, Andrew Hickey, Nicole Pritchard, Per Poulsen, Elizaveta Mitkina Tabaksblat, Britta Weber, Esben Worm, David Pryor, Julie Chu, Nicholas Hardcastle, Jeremy Booth, Val Gebski, Tim Wang, Paul Keall

**Affiliations:** 1 Department of Radiation Oncology, Princess Alexandra Hospital, Brisbane, QLD Australia; 2 The University of Sydney, Sydney, NSW Australia; 3 School of Biomedical Engineering, University of Technology Sydney, Sydney, NSW Australia; 4 ACRF Image X Institute, Sydney, NSW Australia; 5 Department of Radiation Oncology, Crown Princess Mary Cancer Centre, Sydney, NSW Australia; 6 Radiation Physics Laboratory, Sydney Medical School, The University of Sydney, Sydney, NSW Australia; 7 Department of Radiation Oncology, Liverpool-Macarthur Cancer Therapy Centre, Sydney, NSW Australia; 8 Gamma Gurus Pty Ltd, Sydney, NSW Australia; 9 Department of Oncology, Aarhus University Hospital, Aarhus, Denmark; 10 Department of Radiation Oncology, Peter MacCallum Cancer Centre, Melbourne, Victoria Australia; 11 Department of Radiation Oncology, Northern Sydney Cancer Centre, Sydney, NSW Australia; 12 University of Sydney NHMRC Clinical Trials Centre, Sydney, NSW Australia

**Keywords:** Stereotactic radiotherapy, Liver Cancer, Hepatocellular carcinoma, Oligometastases, Kilovoltage intrafraction monitoring, LARK trial

## Abstract

**Background:**

Stereotactic Ablative Body Radiotherapy (SABR) is a non-invasive treatment which allows delivery of an ablative radiation dose with high accuracy and precision. SABR is an established treatment for both primary and secondary liver malignancies, and technological advances have improved its efficacy and safety. Respiratory motion management to reduce tumour motion and image guidance to achieve targeting accuracy are crucial elements of liver SABR. This phase II multi-institutional TROG 17.03 study, **L**iver **A**blative **R**adiotherapy using **K**ilovoltage intrafraction monitoring (LARK), aims to investigate and assess the dosimetric impact of the KIM real-time image guidance technology. KIM utilises standard linear accelerator equipment and therefore has the potential to be a widely available real-time image guidance technology for liver SABR.

**Methods:**

Forty-six patients with either hepatocellular carcinoma or oligometastatic disease to the liver suitable for and treated with SABR using Kilovoltage Intrafraction Monitoring (KIM) guidance will be included in the study. The dosimetric impact will be assessed by quantifying accumulated patient dose distribution with or without the KIM intervention. The patient treatment outcomes of local control, toxicity and quality of life will be measured.

**Discussion:**

Liver SABR is a highly effective treatment, but precise dose delivery is challenging due to organ motion. Currently, there is a lack of widely available options for performing real-time tumour localisation to assist with accurate delivery of liver SABR. This study will provide an assessment of the impact of KIM as a potential solution for real-time image guidance in liver SABR.

**Trial registration:**

This trial was registered on December 7th 2016 on ClinicalTrials.gov under the trial-ID NCT02984566.

**Supplementary Information:**

The online version contains supplementary material available at 10.1186/s12885-021-08184-x.

## Background

Stereotactic Ablative Body Radiation Therapy (SABR) also known as Stereotactic Body Radiation Therapy (SBRT) is a technique used to deliver high-precision, ablative doses of radiation in a small number of fractions to an extra-cranial target [1].

Worldwide, there has been a rapid adoption of SABR to treat a variety of malignancies [2] at a range of sites including lung [3], liver [4] and spine [5]. In the setting of the liver, SABR is an effective and a potentially curative treatment for hepatocellular carcinomas [6] and oligometastatic disease [7].

SABR requires accurate knowledge of the location of a tumour and its physiological motion in relation to surrounding structures. Intrafraction motion can result in geographical inaccuracies in SABR treatment and these, in turn, can compromise treatment outcome and have the potential to increase toxicity. A major challenge for liver SABR is the management of respiratory motion and multiple techniques have been introduced to either proactively manage respiratory motion (e.g. through active breathing control and respiratory gating) or reduce liver motion (e.g. abdominal compression). In liver SABR, implanting radio-opaque fiducial markers around a tumour may allow more accurate and precise localisation of the target volume during treatment delivery. Wahl et al., demonstrated 0% local failure in patients who received liver SABR with fiducial placement to guide treatment delivery [8]. Real-time tumour tracking, intuitively, would also elevate the confidence of precise dose delivery.

**Fig. 1:**
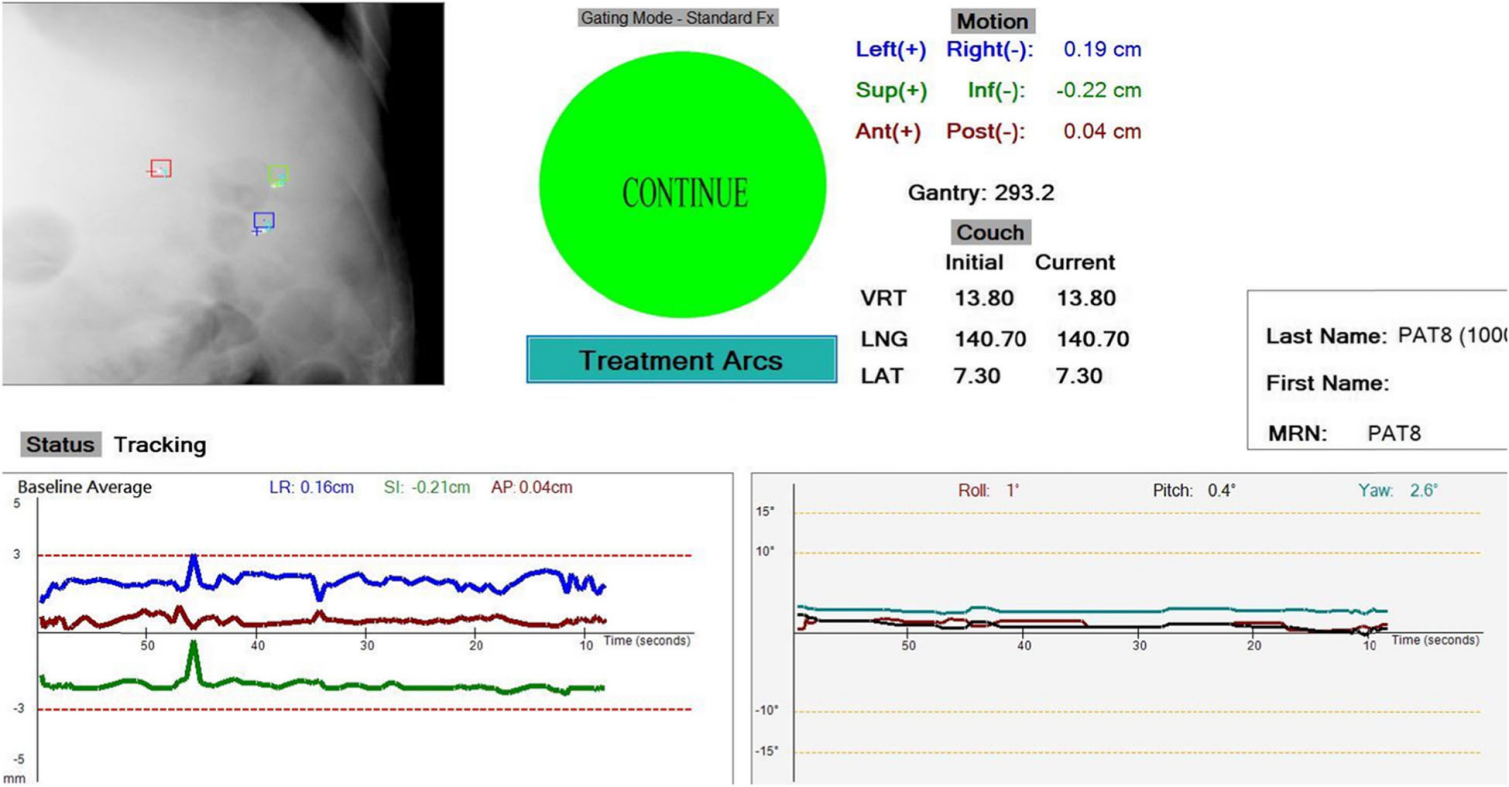
A screenshot of the KIM user interface for liver SABR to be used in the LARK trial. Live-streamed kilovoltage images show the marker positions (crosses) and the planned positions (boxes). From the kilovoltage images the liver 3D translation and 3D rotation values are displayed. If the target motion exceeds a pre-set threshold from the planned position the operator is warned and then instructed to pause the treatment. The patient position is then shifted to align with the beam and the treatment continues

The current Image Guided Radiation Therapy (IGRT) standard of care requires a cone-beam computed tomography (CBCT) scan prior to the beam delivery for localisation followed by appropriate adjustment of the treatment couch and verification CBCT. This process can be repeated between the treatment beams and upon completion of the treatment to ensure accurate patient positioning during treatment. The potential benefits of intra-fraction monitoring are numerous. Without the continuous knowledge of the target position during the beam delivery, inaccurate dose delivery may result in suboptimal disease control and increased toxicities. Kilovoltage Infraction Monitoring (KIM) is an emerging real-time IGRT method. The ability for KIM to achieve real-time imaging to trigger manual pause of the beam delivery when the tumour motion exceeds a pre-set tolerance (Fig. 1) has been clinically investigated for prostate cancer SABR. A phase II study Stereotactic Prostate Adaptive Radiotherapy utilising Kilovoltage intrafraction monitoring (TROG SPARK 15.01) recently demonstrated the clinical benefit of intra-fraction monitoring with KIM guidance in prostate cancer [9]. In a recent computational study exploring the benefit of MLC tracking using KIM guidance in liver SABR, KIM detected much larger intrafraction monitoring than the pre-set tolerance [10], highlighting the importance of future studies in this space. Additionally, a separate study showed that with the application of gating or tracking, more patients are able to receive full isotoxic prescription [11]. The LARK trial will be the first clinical application of KIM outside of the prostate.

There are a range of technologies that provide real-time monitoring of the liver target. Direct image guidance without the need for markers can be achieved using ultrasound [12] or an MRI-Linac [13] 2D position monitoring for liver SABR achievable with markers using Elekta’s XVI or Varian’s Triggered Imaging. 3D position monitoring for liver SABR has been achieved with Calypso (Varian Medical Systems, Palo Alto, CA) [14] and a combined internal-external monitoring system ‘COSMIK’ [15]. Both of the latter studies were from Aarhus University. Their institutional experience of liver SABR using Calypso continuous internal electromagnetic-based gating included 15 patients [14]. This study reported improved geometric and dosimetric accuracy compared with standard treatment. Potentially, KIM has a few practical benefits over the Calypso system. Firstly, KIM uses existing imaging equipment on a standard linear accelerator to achieve intrafraction motion monitoring. Secondly, when simulation MRIs are used for target volume delineation in liver SABR, the standard gold fiducials which are used for KIM are better visualised than the Calypso electromagnetic transponders. Their institutional experience of liver SABR using COSMIK combined x-ray and respiratory signal method to achieve real-time guidance on a standard linear accelerator [16]. COSMIK has been prospectively implemented for liver SABR. With the combined use of the respiratory signal, COSMIK requires fewer x-ray images than KIM and has a lower latency. On the other hand, KIM provides rotation in addition to translation and may have higher accuracy than COSMIK as it only uses the x-ray images.

In addition to higher accuracy in treatment delivery, another potential benefit of using KIM is the reduction in imaging dose and faster treatment time. A retrospective analysis of patients treated with liver SABR at one of our institutions demonstrated an average of 5 CBCTs for each fraction resulting in 25 CBCTs per treatment course which translates to a total effective dose from imaging to be 186 mSv. The final effective dose from using an initial CBCT and KIM has been calculated to be 43 mSv which is a substantial reduction in imaging dose. In liver SABR, CBCT acquisition and analysis takes a significant portion of the treatment duration. Reducing the number of CBCTs could result in an important reduction of overall treatment time. A reduction in treatment time will increase patient throughput and also improve the patient experience, particularly for patients who experience pain in the supine position or are uncomfortable with their arms above their head for long periods of time.

An alternative method to KIM, COSMIK, also uses a combined x-ray and respiratory signal method to achieve real-time guidance on a standard linear accelerator [16]. COSMIK has been prospectively implemented for liver SABR. With the combined use of the respiratory signal, COSMIK requires fewer x-ray images than KIM and has a lower latency. On the other hand, KIM provides rotation in addition to translation and may have higher accuracy than COSMIK as it only uses the x-ray images.

The primary goal of the LARK trial is to assess the dosimetric impact of the KIM real-time IGRT technology, which utilises standard linear accelerator equipment, for liver cancer SABR.

## Methods/design

This study is designed as a multi-institutional single arm phase II study. The LARK trial co-ordination will be conducted by the Trans Tasman Radiation Oncology Group (TROG). Forty-six patients who are eligible for liver SABR treatment with either primary or secondary liver malignancy will be treated with the incorporation of KIM. This study has been approved by Western Sydney Local Health District Research Ethics Board.

Each participating site will be required to undertake a credentialing procedure for review before enrolling any patients. The credentialing process includes the submission of a treatment plan meeting the planning criteria, the completion of the KIM commissioning and quality assurance procedures and an independent review. The KIM commissioning tests were adapted for liver cancer monitoring from Ng et al. [17]. The tests include static tests to ensure coincidence of coordinate systems between KIM and the linear accelerator, dynamic tests with liver patients’ breath-hold and free breathing motion trajectories [15] and treatment interruption tests in which KIM is used to interrupt the treatment. For each test, KIM passes if the mean error of KIM reported results, as compared with the input motion, are less than 1 mm with a standard deviation of less than 2 mm. Ongoing KIM quality assurance tests with the same criteria will be performed throughout the trial at a recommended interval of 1 month.

We aim to test the hypotheses that KIM improves cancer targeting accuracy, patient dose and outcomes. This improvement is defined as the success rate which is the ability of a KIM-corrected patient dose distribution to improve the Planning Target Voume (PTV) dose to 95% of the volume (D_95_) or the liver dose to 50% of the volume (D_50_) by 5% compared to the patient dose distribution without KIM.

This study will accrue patients from five to seven institutions across Australia and one in Denmark. The dataset generated during and/or analysed during the current study are available from the corresponding author on reasonable request.

**Fig. 2:**
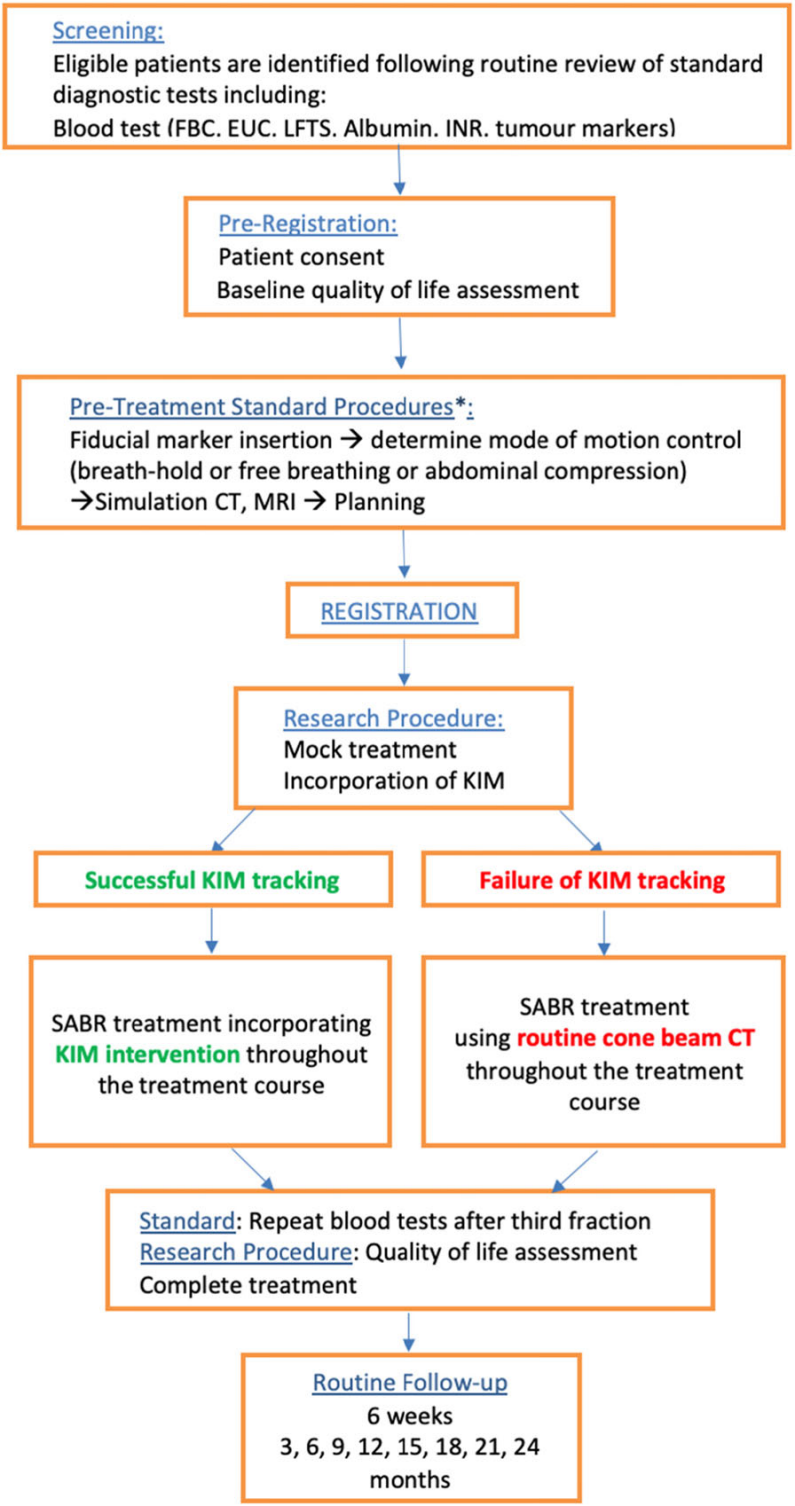
The LARK study schema

The study schema is shown in Fig. 2.

Following each treatment, dosimetric calculations will be performed to compare the dosimetric accuracy of the treatment with KIM incorporation against treatment that would have been delivered in the absence of KIM. Technological assessment of KIM to quantify the clinical practice impact as well as treatment outcome data will be obtained.

### Primary endpoint

The primary outcome is to measure the dosimetric impact of the KIM real-time IGRT technology for liver cancer SABR. A dose accumulation method will be used to determine the dose delivered to the patient with KIM, and also the dose distributions that would have been delivered to the patient had KIM not been used. The isodose distributions and dose volume histograms for each session will be calculated and compared.

### Secondary endpoints

- Assess treatment outcome
  - ○ Local control, toxicity and quality of life
- Quantitative assessment of treatment time, imaging dose, PTV margins, CBCT dose reconstruction
- Qualitative assessment of dose reduction with MLC tracking (simulation study only)

## Key selection criteria

All patients eligible for liver SABR (with fiducial marker placement) for hepatocellular carcinoma or secondary liver malignancy are eligible for this study.

### Inclusion

#### HCC

- Diagnosed by either radiological guidelines (> 1 cm, enhancing arterial phase and wash-out in later phase) or biopsy
- Child–Pugh stage A/B7

#### Liver metastases

- Biopsy preferred but detected on imaging allowed
- Controlled primary tumour: at least 3 months since original tumour treated with curative intent, with no progression at primary site

Patients will be required to be age 18 or over, ECOG performance status 0–2 with life expectancy greater than 6 months. Three or fewer liver lesions, lesion size less than 10 cm in the largest diameter in any direction for a single lesion (and up to 10 cm cumulative diameter for multiple lesions), unsuitable for RFA/MWA. Patients may have had previous surgery, RFA/MWA or ethanol injection, or TACE. All blood work obtained within 6 weeks prior to study entry with adequate organ function. Patients must have been discussed at a multidisciplinary tumour board with the consensus opinion for SABR.

### Exclusion

Patients with hepatocellular carcinoma who have evidence of metastatic disease including nodal or distant metastases, patients with metastatic disease who have had complete liver disease response to first-line chemotherapy (i.e. no target for SABR). Previous radiation to the liver (including SIRTEX), untreated HIV or active hepatitis B, systemic antineoplastic drug therapy within 7 days before inclusion, pregnant or lactating women.

Patients with less than two fiducial markers and/or fiducial markers with greater than 10 cm will be excluded.

## Radiotherapy planning and treatment

### Pre-treatment

Fiducial markers are mandatory for this study. The recommended number of fiducial markers is three or more to allow triangulation and measurement of position in different planes. Fiducials should be inserted 1–2 cm from the tumour to be viable surrogates. To serve a purpose as a better surrogate than using anatomical landmarks, Seppenwoolde et al., recommended the placement of the makers closer than 8 cm to the tumour centre [18]. Fiducial markers should be implanted at least 1 day prior to simulation.

### Simulation

Prior to CT simulation, it is recommended that patients undergo a motion management assessment such as fluoroscopy to determine the best choice of motion management. Assessment could include end-expiration breath-hold (EEBH), deep inspiration breath-hold (DIBH), and free-breathing (FB) with or without abdominal compression. EEBH is the preferred motion management method.

A triple phase CT with contrast and a non-contrast CT should be obtained using maximum 3 mm thickness slices (1 mm preferred). For patients who are suitable for EEBH or DIBH, all scans should be done at breath-hold. A 4DCT should be acquired if free-breathing with or without abdominal compression is utilised to determine tumour motion.

Contrast enhanced MRI can be used to tumour delineation preferably in treatment position on a flat couch top utilising the same motion management method as for the planning CTs. PET-CT may be used for patients with liver metastases.

## Treatment planning

### Target volumes

Using non-contrast CT as the primary dataset, the target volumes are defined as:

- Gross Tumour Volume (GTV) = tumour visible on CT and/or MRI, ideally after review with diagnostic radiologist and consideration of other imaging available (e.g. PET scan for liver metastases).
- Clinical Target Volume (CTV) = GTV
- Internal Target Volume (ITV) is optional but should be established if the patient is to be treated with free breathing or abdominal compression techniques. The magnitude of motion may be determined by measuring the trajectory of the fiducial markers or other surrogate on a 4DCT scan.
- Planning Target volume (PTV) = CTV + 5–15 mm depending on motion management strategy. For treatment using EEBH or DIBH, typically 5 mm in radial directions, and 5–7 mm in cranio-caudal directions from the CTV. Note that the margin can be different in all planes. For treatment using free-breathing, typically 5 mm in all direction from the ITV.

### Dose prescription

The range of dose prescriptions and fractionation schedules including the planning dose constraints are adapted from existing recommendations [19–21] and clinical trials including UK CORE, RTOG 1112, NRG BR001, agreed upon by radiation oncologists from six institutions across Australia and Denmark with expertise in liver SABR.

#### Hepatocellular carcinoma

Six dose prescriptions (50 Gy, 45 Gy, 40 Gy, 35 Gy, 30 Gy or 27.5 Gy) in five fractions depending on the clinical scenario and the radiation oncologist choice. Interfraction time should be between 24 and 72 h and treatment should be delivered over 5–15 days.

#### Liver metastases

Two fractionation regimens are available depending on the clinical scenario and the radiation oncologist choice. Generally, three fractions are preferred. For three fraction regimens, the dose prescription levels include 54Gy, 51 Gy, 48 Gy, 45 Gy or 42 Gy delivered on alternate days over 5–7 days. For five fraction regimens, the dose prescription levels are 60 Gy, 55 Gy, 50 Gy, 45 Gy 40 Gy delivered over 10–15 days with a preferred interfraction interval greater than 36 h.

### Treatment techniques

Volumetric Modulated Arc Radiotherapy (VMAT) or IMRT (Intensity Modulated Radiation Therapy) planning is required and flattening filter free beams are allowed. A real-time pre-treatment quality assurance technical review will be performed via TROG for all participants. Target volume planning goals outlined in Table 1 and organ at risk planning guidelines are provided in the LARK Radiation Therapy Quality Assurance document. The planning dose-volume constraints (supplementary Tables 1–4 are appended to this manuscript).

**Table 1:** Target volume doses for the TROG 17.03 LARK clinical trial. TD = Target Dose (prescribed dose). RVR = Remaining Volume at Risk

LARK Target Volume Doses							
Standardised Name	Constraint	Per Protocol		Minor Variation		Major Variation	
		PTVs around GTVs	PTVs around Non-GTV CTVs^**a**^	PTVs around GTVs	PTVs around Non-GTV CTVs^**a**^	PTVs around GTVs	PTVs around Non-GTV CTVs^**a**^
GTV	D100%	≥100% of the TD		95–100% of the TD		≤95% of the TD	
PTV	Dmax (0.03 cc)	120–140% of the TD		140–150% of the TD or 110–120% of the TD		≥150% of the TD or ≤ 110% of the TD	
	D95%	95–105% of the TD		90–95% or 105–110% of the TD, and > 25 Gy	85–95% or 105–115% of the TD, and > 25 Gy	≤90% or ≥110% of the TD, or ≤ 25 Gy	≤85% or ≥115% of the TD, or ≤ 25Gy
RVR	Dmax (0.03 cc)	≤120% of the TD		–		≥120% of the TD	

^a^ Non-GTV CTVs represents regions at high risk for microscopic disease, including non-tumour vascular thrombi, prior TACE sites, or adjacent RFA or other ablation sites. Treatment of these high-risk sites are permitted

#### Treatment delivery

Each participant will undergo a pre-treatment assessment session prior to the first fraction to assess feasibility of KIM. During this pre-treatment session, a CBCT scan will be acquired. KIM will be used to track the fiducials on the projection images during at least 2 CBCT acquisitions. KIM reported 3D positions will be compared with manual fiducial matching by the radiation therapists on the reconstructed CBCT. If the KIM reported couch shift is 2 mm or less from the manual match couch shift, the treatment course will commence with KIM. In scenarios where KIM fails to track the fiducial markers, or the KIM reported couch shift is more than 2 mm from the manual match the current standard-or-care IGRT with CBCT will be utilised for the patient treatment.

During patient treatments, following the initial CBCT and patient repositioning, KIM will be used to provide continuous monitoring of the tumour motion during treatment beam-on. If using breath-hold technique, fiducial displacement of 3 mm from the baseline for more than 5 s, the beam will be manually turned off. If the fiducial does not return to baseline for two consecutive breath-holds, the patient will be realigned by shifting the couch. For free-breathing or abdominal compression technique, if the fiducials exceed the ITV-based threshold for more 5 s the beam will be turned off until the fiducial markers return within tolerance. If the fiducials exhibit a baseline shift, the patient will be realigned with a couch shift.

For both breath-hold and free-breathing treatments, participating institutions are permitted and encouraged to use their clinical standard motion management techniques including optical devices such as Real-time Position Management (RPM, Varian, Palo Alto, CA, USA), Active Breathing Coordinator (ABC, Elekta, Stockholm, Sweden) or AlignRT (VisionRT, London, UK).

#### Assessments

##### Dosimetric / technological assessment

For each fraction, the targeting accuracy and delivered patient dose distribution will be determined via paired control by comparing the measured targeting error and dose with KIM to those that would have been delivered in the absence of KIM.

We will also perform a technology assessment of KIM to quantify the clinical practice impact by:

1. Quantifying the impact on workflow using KIM through time-motion studies
2. Evaluating operator and clinician confidence in KIM’s reliability and clinical efficacy through a technology-impact survey
3. Quantifying the system robustness through hardware and software fault reporting
4. Performing system quality assurance (QA), at multiple sites, sharing the results through a web-based upload and provide feedback for QA improvement

##### Acute / late toxicity and disease outcome assessment

Patients will undergo clinical assessment as well as routine blood tests, imaging at pre-defined time points as outlined in Fig. 2. Both acute and late toxicity will be reported using NCI Common Terminology Criteria for Adverse Events (CTCAE) version 4 and Radiation Induced Liver Disease (RILD).

Treatment response will be assessed by triple-phase CT or MRIs for patients with HCC. For patients with metastatic liver disease, local control will be assessed with CT scan and /or PET CT or MRI. The preferred assessment for HCC is the modified RECIST criteria [22] and for metastatic disease RECIST and/or PERCIST criteria [23].

Patient-reported outcomes (PROs) will be assessed using the general EORTC QLQ-C30 and disease specific quality of life questionnaires EORTC QLQ-HCC18.

In this study, 46 patients are needed to test the hypothesis that KIM can improve patient dose distribution.

We consider a treatment a success if the KIM-corrected patient dose distribution improves the planning target volume (PTV) dose to 95% of the volume (D_95_) or the liver dose to 50% of the volume (D_50_) by 5% compared to dosimetry without KIM. A dose accumulation method [24] will be used to determine the efficacy of KIM, where the isodose distributions and dose volume histograms for each treatment course will be calculated with KIM corrections as treated, and estimated without KIM corrections.

A 60% or higher treatment success rate would suggest KIM is promising and worthy of further investigation. Alternatively, a treatment success rate of 40% or lower would suggest than KIM should not be considered for this treatment modality. Using Simon’s two-stage optimum design [25], a sample size of 46 patients will have 80% power with 95% confidence to rule out a 40% success rate in favour of a more interesting success rate of 60%.

##### Interim analysis for futility

The success rate will be determined after 16 patients have been accrued and completed treatment. If fewer than nine successes are observed, consideration will be given to re-examining the cause of the lack of success and the trial could be re-designed or stopped for futility at this point. However, recruitment will continue while this futility boundary is evaluated. If nine or more success are observed, 30 additional patients will be accrued for a total of 46 patients. The main analysis will be performed after at least 46 patients have been followed for 2 years, or the data is sufficiently mature to report the results earlier.

Exploratory analyses comparing outcomes of the KIM ‘failures’ to KIM ‘successes’ will also be performed to obtain estimates of whether the degree of accuracy afforded by KIM actually translates to clinical endpoints.

## Discussion

This multi-centre study aims to accrue 46 patients to test the hypothesis that KIM provides a real-time IGRT solution to improve cancer targeting accuracy, dose delivery and treatment outcomes in patients receiving liver SABR. Several studies have demonstrated liver motion up to several centimetres during treatment [15, 26, 27] and a retrospective review at our institution also demonstrated significant intrafraction motion which would not have been observed without using a real-time imaging system such as KIM.

Other potential benefits of KIM technology include a reduction in imaging radiation dose and overall treatment time compared to current IGRT method of using CBCT. We have demonstrated that continuous kilovoltage imaging results in substantially less imaging dose than multiple CBCT scans. CBCTs are acquired during breath-holds, which is often the reason for long treatment times leading to patient fatigue and instability in the patients’ ability to maintain consistent breath-holds.

The liver is a radiosensitive organ, and particularly in patients with liver cirrhosis, maximum sparing of the functioning liver is a critical goal in SABR. Continuous intrafraction monitoring has the potential to improve the accuracy of treatment delivery for better disease outcome and reduced treatment-related toxicity. Ultimately, if direct intra-fraction visualisation of the target lesion using KIM method is successful, it may help to safely reduce the PTV margins to maximise further liver sparing and minimise toxicity.

## Supplementary Information

**Additional file 1.**

## Data Availability

The LARK Trial Management Committee support the use of the data acquired in this study for further research within the bounds of patient privacy and ethics concerns. The protocol states: *De-identified data collected in this study will be stored perpetually and may be used for future research. The stored data will only be used in future research under the approval of a Human Research Ethics Committee*. Researchers interested in the use of the LARK trial data should contact the study authors.

## Abbreviations

CBCT: Cone-Beam Computed Tomography
CTV: Clinical Tumour Volume
DIBH: Deep Inspiration Breath-Hold
EEBH: End-Exhale Breath-Hold
GTV: Gross Tumour Volume
IGRT: Image Guided Radiation Therapy
IMRT: Intensity Modulated Radiotherapy
ITV: Internal Target Volume
KIM: Kilovoltage Intrafraction Monitoring
LARK: Liver Ablative Radiotherapy using Kilovoltage intrafraction monitoring
MLC: Multileaf Collimator
PERCIST: Positron Emission Tomography Response Criteria In Solid Tumours
PTV: Planning Target Volume
QA: Quality Assurance
RECIST: Response Evaluation Criteria In Solid Tumours
RILD: Radiation Induced Liver Disease
SABR: Stereotactic Ablative Body Radiotherapy
SBRT: Stereotactic Body Radiation Therapy
VMAT: Volumetric Modulated Arc Therapy
