# Supplementary material for: Study protocol of the LARK (TROG 17.03) clinical trial: a phase II trial investigating the dosimetric impact of Liver Ablative Radiotherapy using Kilovoltage intrafraction monitoring
Source: BMC Cancer. 2021 May 3;21:494. doi: 10.1186/s12885-021-08184-x (PMC8091536; doi:10.1186/s12885-021-08184-x)
Supplement: Supplementary file 1 — Additional file 1. [file 12885_2021_8184_MOESM1_ESM.docx]

Supplementary Table 1. Organ at Risk Dose Constraints Hepatocellular Carcinoma (HCC): 27.5 – 50 Gy in 5 fractions.

| **Standardised Name** | **Constraint** | **Hepatocellular Carcinoma (HCC):**  **27.5 – 50 Gy in 5 fx** | | |
| --- | --- | --- | --- | --- |
|  | | **Acceptable** | **Minor Violation** | **Major Violation** |
| **Aorta** | D0.5cc | ≤53Gy | - | >53Gy |
| **CHT** | D21cc | <40Gy | ≥40Gy | - |
|  | D24cc | <37.7Gy | ≥37.7Gy | - |
|  | D37cc | <26Gy | ≥26Gy | - |
|  | D45cc | <21Gy | ≥21Gy | - |
|  | Dmean | <19Gy | ≥19Gy | - |
| **Chestwall** | D0.5cc | ≤39Gy | >39Gy | - |
|  | D30cc | ≤32Gy | >32Gy | - |
| **CBD** | D0.03cc | ≤41Gy | >41Gy | - |
| **Duodenum** | D0.5cc | ≤30Gy | >30Gy but ≤32Gy | >32Gy |
|  | D1cc | ≤33Gy | >33Gy | - |
|  | D5cc | ≤25Gy | >25Gy |  |
|  | D9cc | ≤15Gy | >15Gy |  |
|  | D10cc | ≤25Gy | - | >25Gy |
| **Gallbladder** | D0.5cc | ≤55Gy | >55Gy | - |
| **Heart** | D0.03cc | ≤38Gy | - | >38Gy |
|  | D0.5cc | ≤29Gy | >29Gy but ≤35Gy | >35Gy |
|  | D5cc | ≤30Gy | - | >30Gy |
| **V_CavaInf** | D0.5cc | ≤53Gy | - | >53Gy |
| **Kidney_R, Kidney_L, Kidneys** | Dmean | ≤10Gy | >10Gy but ≤12Gy | >12Gy |
|  | V16Gy | Record only | - |  |
| **Kidney_R, Kidney_L, (use if patient has solitary kidney or Kidney_L or Kidney_R Dmean >10Gy)** | V10Gy | ≤10% | >10% but ≤45% | >45% |
| **LargeBowel** | D0.5cc | ≤32Gy | - | >32Gy |
| **BilatLung** | D0.03cc | ≤120% TD | >120% TD but ≤140% TD | >140% TD |
| **Oesophagus** | D0.5cc | ≤32Gy | >32Gy but ≤34Gy | >34Gy |
| **RVR** | D0.03cc | ≤120% TD | - | >120% TD |
| **Skin** | D0.5cc | ≤32Gy | >32Gy but ≤39.5Gy | >39.5Gy |
|  | D10cc | ≤36.5Gy | - | >36.5Gy |
| **SmallBowel** | D0.5cc | ≤30Gy | >30Gy but ≤32Gy | >32Gy |
|  | D5cc | ≤25Gy | >25Gy | - |
|  | D10cc | ≤11.4Gy | - | >11.4Gy |
| **SpCord_PRV** | D0.03cc | <28Gy | - | >28Gy |
|  | D0.1cc | Record only | - |  |
|  | D0.5cc | ≤25Gy | >25Gy | - |
|  | D1.2cc | Record only | - |  |
| **Stomach** | D0.5cc | ≤30Gy | >30Gy but ≤32Gy | >32Gy |
|  | D5cc | ≤25Gy | >25Gy | - |
|  | D10cc | ≤25Gy | - | >25Gy |
|  | D50cc | ≤12Gy | >12Gy | - |

Supplementary Table 2. Organ at Risk Dose Constraints Liver Metastases (METS) 3 fractions: 42 - 54 Gy in 3 fractions.

| **Standardised Name** | **Constraint** | **Liver Metastases (METS)**  **3 fx: 42 - 54 Gy in 3 fx** | | |
| --- | --- | --- | --- | --- |
|  | | **Acceptable** | **Minor Violation** | **Major Violation** |
| **Aorta** | D0.5cc | ≤45Gy | - | >45Gy |
| **CHT** | D21cc | <33.8 | ≥33.8Gy | - |
|  | D24cc | <32Gy | ≥32Gy | - |
|  | D37cc | Record only | - | - |
|  | D45cc | Record only | - | - |
|  | Dmean | Record only | - | - |
| **Chestwall** | D0.5cc | ≤37Gy | >37Gy | - |
|  | D30cc | ≤30Gy | >30Gy | - |
| **CBD** | D0.03cc | ≤36Gy | >36Gy | - |
| **Duodenum** | D0.5cc | ≤22.2Gy | - | >22.2Gy |
|  | D1cc | Record only | - | - |
|  | D5cc | ≤16.5Gy | - | >16.5Gy |
|  | D9cc | Record only | - | - |
|  | D10cc | ≤11.4Gy | - | >11.4Gy |
| **Gallbladder** | D0.5cc | Consider using 5# regimen if gallbladder in proximity | | |
| **Heart** | D0.03cc | Record only | - | - |
|  | D0.5cc | ≤24Gy | >24Gy but ≤26Gy | >26Gy |
|  | D5cc | Record only | - |  |
| **V_CavaInf** | D0.5cc | ≤45Gy | - | >45Gy |
| **Kidney_R, Kidney_L, Kidneys** | Dmean | Record only | - |  |
|  | V16Gy | ≤200cc | - | >200cc |
| **Kidney_R, Kidney_L, (use if patient has solitary kidney or Kidney_L or Kidney_R Dmean >10Gy)** | V10Gy | Record only | - | - |
| **LargeBowel** | D0.5cc | ≤28.2Gy | - | >28.2Gy |
| **BilatLung** | D0.03cc | ≤120% TD | >120% TD but ≤140% TD | >140% TD |
| **Oesophagus** | D0.5cc | ≤25.2Gy | - | >25.2Gy |
| **RVR** | D0.03cc | ≤120% TD | - | >120% TD |
| **Skin** | D0.5cc | ≤32Gy | >32Gy | - |
|  | D10cc | ≤30Gy | >30Gy | - |
| **SmallBowel** | D0.5cc | ≤25.2Gy | - | >25.2Gy |
|  | D5cc | ≤17.7Gy | - | >17.7Gy |
|  | D10cc | Record only | - | - |
| **SpCord_PRV** | D0.03cc | ≤20Gy | - | >20Gy |
|  | D0.1cc | ≤18Gy | >18Gy | - |
|  | D0.5cc | Record only | - | - |
|  | D1.2cc | ≤12.3Gy | - | - |
| **Stomach** | D0.5cc | ≤22.2Gy | - | >22.2Gy |
|  | D5cc | Record only | - | - |
|  | D10cc | ≤16.5Gy | - | >16.5Gy |
|  | D50cc | Record only | - | - |

Supplementary Table 3. Organ at Risk Dose Constraints Liver Metastases (METS) 5 fractions: 40 - 60 Gy in 5 fractions.

| **Standardised Name** | **Constraint** | **Liver Metastases (METS)**  **5 fx: 40 - 60 Gy in 5 fx** | | |
| --- | --- | --- | --- | --- |
|  | | **Acceptable** | **Minor Violation** | **Major Violation** |
| **Aorta** | D0.5cc | ≤53Gy | - | >53Gy |
| **CHT** | D21cc | <40Gy | ≥40Gy | - |
|  | D24cc | <37.7Gy | ≥37.7Gy | - |
|  | D37cc | <26Gy | ≥26Gy | - |
|  | D45cc | <21Gy | ≥21Gy | - |
|  | Dmean | <19Gy | ≥19Gy | - |
| **ChestWall** | D0.5cc | ≤39Gy | >39Gy | - |
|  | D30cc | ≤32Gy | >32Gy | - |
| **CBD** | D0.03cc | ≤41Gy | >41Gy | - |
| **Duodenum** | D0.5cc | ≤30Gy | >30Gy but ≤32Gy | >32Gy |
|  | D1cc | ≤33Gy | >33Gy | - |
|  | D5cc | Record only | - | - |
|  | D9cc | ≤15Gy | >15Gy | - |
|  | D10cc | ≤25Gy | - | >25Gy |
| **Gallbladder** | D0.5cc | ≤55Gy | >55Gy | - |
| **Heart** | D0.03cc | ≤38Gy | - | >38Gy |
|  | D0.5cc | ≤29Gy | >29Gy but ≤35Gy | >35Gy |
|  | D5cc | ≤30Gy | - | >30Gy |
| **V_CavaInf** | D0.5cc | ≤53Gy | - | >53Gy |
| **Kidney_R, Kidney_L, Kidneys** | Dmean | ≤10Gy | >10Gy but ≤12Gy | >12Gy |
|  | V16Gy | Record only | - | - |
| **Kidney_R, Kidney_L, (use if patient has solitary kidney or Kidney_L or Kidney_R Dmean >10Gy)** | V10Gy | ≤10% | >10% but ≤45% | >45% |
| **LargeBowel** | D0.5cc | ≤32Gy | - | >32Gy |
| **BilatLung** | D0.03cc | ≤120% TD | >120% but ≤140% TD | >140% TD |
| **Oesophagus** | D0.5cc | ≤32Gy | >32Gy but ≤34Gy | >34Gy |
| **RVR** | D0.03cc | ≤120% TD | - | >120% TD |
| **Skin** | D0.5cc | ≤32Gy | >32Gy but ≤39.5Gy | >39.5Gy |
|  | D10cc | ≤36.5Gy | >36.5Gy | - |
| **SmallBowel** | D0.5cc | ≤30Gy | >30Gy but ≤32Gy | >32Gy |
|  | D5cc | ≤25Gy | >25Gy | - |
|  | D10cc | ≤11.4Gy | - | >11.4Gy |
| **SpCord_PRV** | D0.03cc | <28Gy | - | >28Gy |
|  | D0.1cc | Record only | - | - |
|  | D0.5cc | ≤25Gy | >25Gy | ~~-~~ |
|  | D1.2cc | Record only | - | - |
| **Stomach** | D0.5cc | ≤30Gy | >30Gy but ≤32Gy | >32Gy |
|  | D5cc | ≤25Gy | >25Gy | - |
|  | D10cc | ≤25Gy | - | >25Gy |
|  | D50cc | ≤12Gy | >12Gy | - |

Supplementary Table 4. Liver dose recommendations.

|  | **Constraint** | **Description** | **Prescription Dose** | **Acceptable** | **Minor Violation** | **Major Violation** |
| --- | --- | --- | --- | --- | --- | --- |
| **HCC** | Liver-GTV Dmean | Dmean  Mean dose to liver minus GTV (Child-Pugh Classification_A (CP_A)) | 50Gy | ≤13Gy | >13Gy but ≤13.2Gy | >13.2Gy |
|  |  |  | 45Gy | ≤15Gy | >15Gy but ≤15.2Gy | >15.2Gy |
|  |  |  | 40Gy | ≤15Gy | >15Gy but ≤15.2Gy | >15.2Gy |
|  |  |  | 35Gy | ≤15.5Gy | >15.5Gy but ≤15.7Gy | >15.7Gy |
|  |  |  | 30Gy | ≤16Gy | >16Gy but ≤16.2Gy | >16.2Gy |
|  |  |  | 27.5Gy | ≤17Gy | ≤17.2Gy |  |
|  | Liver-GTV Dmean | Dmean  Mean dose to liver minus GTV (Child-Pugh Classification_B7 (CP_B7)) | Prescription dose determined by the Liver-GTV mean dose tolerance | ≤5Gy | >5Gy but ≤6Gy | >6Gy |
|  | Liver-GTV | V10Gy | - | ≤70% | >70% | - |
| **Mets 3 fx** | Liver-GTV | DMean* | - | ≤15Gy | >15Gy | - |
|  |  | D50% | - | <15Gy | ≥15Gy | - |
| **Mets 5 fx** | Liver-GTV | Dmean* | - | ≤15.2Gy | - | >15.2Gy |
|  |  | V10Gy | - | ≤70% | >70% | - |
| **HCC** | Liver-15Gy | Volume of liver receiving less than 15Gy | - | ≥800 cc | <800cc but ≥ 700cc | <700cc |
| **Mets 3 fx and 5 fx** |  |  | - | ≥700 cc | <700cc | - |

*If CP_B7 prescription dose determined by the Liver-GTV mean dose tolerance (acceptable mean dose ≤5Gy).
